# Comparison of predictive value of NT-proBNP, sST2 and MMPs in heart failure patients with different ejection fractions

**DOI:** 10.1186/s12872-020-01493-2

**Published:** 2020-04-30

**Authors:** Wei Pan, Donghui Yang, Peng Yu, Huizhen Yu

**Affiliations:** 1 Key laboratory of Geriatrics, Shengli Clinical Medical College of Fujian Medical University, Fujian Institute of Clinical Geriatrics, No.134, Road Dongjie, District Gulou, Fuzhou, 350001 P.R. China; 2 Department of cardiology, The Eighth Affiliated Hospital, Sun Yat-Sen University, Shenzhen, 518000 China; 3 Zhengzhou People’s Hospital, Zhengzhou, 450000 China; 4 Department of medicine, Fujian Provincial Hospital South Branch, Fuzhou, 350028 China

**Keywords:** HF, sST2, MMPs, NT-proBNP, Predictive value

## Abstract

**Background:**

This study sought to compare the predictive value of NT-proBNP, sST2 and MMPs in HF with different ejection fractions from a population in southern China.

**Methods:**

A cross-sectional study was conducted on 113 HF patients admitted to Fujian Provincial Hospital from December 2016 to March 2018.The patients were divided into three subgroups: 60 cases in HFpEF group (LVEF≥50%), 28 cases in HFmrEF group (41% ≤ LVEF≤49%) and 25 cases in HFrEF group (LVEF≤40%). ELISA method was applied to detect the concentrations of sST2, MMP-2 and MMP-9. Electrochemical luminescence immunoassay was applied to detect the concentration of plasma NT-proBNP. Univariate and multivariate Cox and logistic regression models were used to analyze the diagnostic significance of these plasma biomarkers in HF patients. Kaplan–Meier survival curves were used to assess the prognostic value of sST2 in the incidence of long-term adverse events during study.

**Results:**

This study showed that plasma sST2 levels in HFrEF or HFmrEF patients were significantly higher than in HFpEF patients. Plasma levels of MMP-2 and MMP-9 in HFrEF patients were apparently higher than in HFpEF or HFmrEF patients. For the diagnosis of HFpEF, the AUC of NT-proBNP was higher than that of sST2, MMP-2 and MMP-9, which were 0.881, 0.717, 0.705 and 0.597, respectively. For the diagnosis of HFmrEF, the AUC of plasma sST2 was higher than that of MMP-2, MMP-9 and NT-proBNP, which were 0.799, 0.678, 0.676 and 0.793, respectively. For the diagnosis of HFrEF, the AUC of plasma NT-proBNP, sST2, MMP-2, and MMP-9 were 0.945, 0.820, 0.814, and 0.774 respectively. Spearman correlation analysis showed that plasma sST2 levels were significantly correlated with plasma MMP-2, MMP-9 and NT-proBNP levels. Further logistic regression analysis showed that except MMP-9, the biomarkers sST2 (OR = 1.960), MMP-2 (OR = 0.805) and NT-proBNP (OR = 0.002) were all independent risk factors for patients with heart failure. Survival analysis results suggested that for patients with HFmrEF, a higher level of plasma sST2 (≥ 0.332 ng/ml at admission) may predict a higher risk of endpoint events and a lower survival rate (*P* < 0.025).

**Conclusions:**

The circulating biomarkers sST2, MMP-2 and NT-proBNP were all independent risk factors for patients with heart failure. The sST2 can be a useful biomarker with both diagnostic and prognostic value in patients with HFmrEF. The higher sST2 level in patients with heart failure was related to a higher incidence of combined endpoint outcome.

## Background

China is stepping into an aging society at present. The prevalence of heart failure (HF) become a serious problem due to the aging of population and the increased survival rate of patients with cardiovascular diseases. HF is a growing epidemic problem because of the significant morbidity and mortality rate [1]. At present, clinical diagnosis of HF is mainly based on medical history, physical signs and auxiliary examinations. Though in recent years, studies have been published on the aid of certain biomarkers in the diagnosis of HF, no ideal biomarker have been established to be widely used in clinical practice. In clinical practice, the plasma level of N-terminal of the prohormone brain natriuretic peptide (NT-proBNP) is considered to be a good reference in the diagnosis and prognosis of HF with reduced ejection fraction (HFrEF). However, NT-proBNP is susceptible to many non-cardiac factor [2], which presented to have some limitations in the diagnosis of HF with mid-range ejection fraction (HFmrEF) and HF with preserved ejection fraction (HFpEF) [3]. Therefore, it is particularly important to explore better biomarkers with high sensitivity and specificity for HFmrEF and HFpEF.

Recently, soluble suppression of tumorigenicity 2 (sST2), matrix metalloproteinase (MMP)-2 and MMP-9 which are associated with cardiac remodeling and tissue fibrosis were reported to apply for the risk assessment in HF patients from western countries. Therefore, sST2 is considered to be the most valuable biomarker after NT-proBNP in HF stratification recommended by guidelines [4, 5]. However, there is still few comparative study on the diagnostic value of sST2, MMPs and NT-proBNP in Asian HF patients with different ejection fraction. So our study was to analyze the characteristic and clinical significance of these circulating blood biomarkers sST2, MMPs and NT-proBNP in HFpEF, HFmrEF and HFrEF patients. By comparing the diagnostic value of all these biomarkers, we may provide a more selective and effective detection for HF patients with different ejection fraction in clinical application.

## Methods

### Study population

A total of 163 consecutive patients with cardiac dysfunction who were hospitalized in Fujian Provincial Hospital from December 2016 to March 2018 were screened to be enrolled in the present study, including 85 males and 78 females, aged 42–86 years old. 163 patients were classified into four groups according to current ACC/AHA guidelines [6]: 50 cases in NYHA class I group, 24 cases in NYHA class II group, 53 cases in NYHA class III group, and 36 patients in NYHA class IV group. According to the standard of left ventricular ejection fraction measured by Cardiac Doppler ultrasound [3], 113 patients in NYHA class from II-IV grade were further divided into three subgroups: 60 cases with HFpEF group (LVEF≥50%), 28 cases with HFmrEF group (41 ≤ LVEF≤49%), 25 cases with HFrEF group (LVEF≤40%). General data such as age, gender, body mass index, blood pressure, blood lipids and renal function were collected. After admission, they were given conventional anti-heart failure medications. All subjects signed the informed consent. The patients who suffered from severe infection, pulmonary embolism, stroke, acute trauma, autoimmune diseases, hematopoietic diseases, malignant tumors, rheumatism, connective tissue diseases, pregnancy and other diseases affecting the secretion of sST2, MMP-2, MMP-9, and mental diseases or with the incomplete data affects the judge were excluded.

### Measurement of various indicators

Height, weight, and blood pressure were measured in all subjects and body mass index (BMI) was calculated. 5 ml of fast median cubital venous blood samples were collected the morning after admission. Sodium, blood sugar, blood lipids, and renal functions were measured by fiduciary institutions in our hospital.

### Echocardiographic data

The routine color echocardiography was performed by the ultrasound specialist in the ultrasound room of Fujian Provincial Hospital. The patients were placed in the left lateral position, the probe was placed on the apex cordis, 2-dimensionally directed left ventricular (LV) M-mode dimensions were acquired from the parasternal long axis and carefully obtained perpendicular to the LV long axis and measured at the level of the mitral valve leaflet tips at end-diastole following the recommendations of the American Society of Echocardiography. LV end-systolic volume and LV ejection fraction (LVEF) were calculated using modified Simpson’s method. Diastolic function was assessed by 2D and Doppler methods. From the apical four chamber view with color flow imaging, the indoor diameter and left ventricular end-diastolic volume, interval and thickness of the left and right ventricular wall and movement were observed. The structure of each valve was observed and the blood flow spectrum in the diastolic period, the acceleration and deceleration time of E wave and a ratio of E wave and A wave (E/A ratio), and the peak early diastolic flow velocity (E), maximum speed of E and peak late diastolic flow velocity (A) were recorded from the mitral valve inflow velocity curve using pulsed wave Doppler at the tips of the mitral valve leaflet. The above measurements are the average of three cardiac cycles’ measurements.

### Detection methods and procedures of main observation indicators

The 5 ml of fasting venous blood samples from subjects were collected in the Ethylene Diamine Tetraacetic Acid (EDTA)-K2 anti-coagulation tube on the next day after admission. The samples were centrifuged at 3000 r/min for 15 min, and the plasma was extracted and transferred The 5 ml of fasting venous blood samples from subjects were collected in the EDTA-K2 anti-coagulation tube on the next day after admission. The samples were centrifuged at 3000 r/min for 15 min, and the plasma was extracted and saved in the − 70°Crefrigerator. Samples were tested at the same time: sST2 kit was purchased from Wuhan Boshide Biological Company (No. EK1116), MMP-2 and MMP-9 kits were purchased from Hailian Biological Company (No. m19027652, m19026201). The plasma NT-proBNP concentration was determined by electrochemical luminescence immunoassay. The instrument was Elecsys 2010 (No. SLS-105) from Roche, Japan, and the experiment was completed by the Laboratory of Fujian Provincial Hospital. The indicators were tested strictly in accordance with the operating procedures of the kit instructions.

### Follow up

During the following 1–3 years after discharge, patients were followed up by telephone to record adverse events, including all-cause death, re-admission for heart failure, and combined endpoint for heart failure. All-cause death is defined as death due to cardiovascular events (acute myocardial infarction, stroke, cardiogenic shock, etc.) or non-cardiovascular events (tumor, trauma, respiratory failure, etc.) Re-admission for heart failure was defined as a patient hospitalized for HF or spent more than 24 h in the emergency room. Some patients who were lost to follow-up were defined as the patient who was unable to find the ID number or the ID number was wrong, whose data were treated as the censored value in the final survival analysis.

### Statistical methods

All data were processed by using SPSS 22.0 statistical software. Descriptive analyses are presented as mean ± standard deviation for variables with normal distribution. The variables with non-normal distribution were expressed as the median (interquartile range). Study groups were compared using the Mann-Whitney U test and the Kruskal-Wallis H test for data that did not present normal distribution, and the Nemenyi method was used for further comparison between two groups. Categorical variables were compared using chi-square tests. The receiver operating characteristic curve (ROC) was drawn to calculate the area under the curve (AUC) to evaluate the diagnostic value of the marker for heart failure. Correlation analysis between variables, continuous variables that presented normal distribution were analyzed by Pearson correlation, and variables that did not present normal distribution were analyzed by Spearman correlation. Spearman correlation analysis was used to describe the correlation between indicators. Logistic regression was used to analyze the risk factors for heart failure. The Kaplan–Meier survival curve was used to assess the incidence of adverse events in patients.

## Results

### Basic data of HF patients

113 patients were enrolled in this study. The average age of the participants was.

69.92 ± 13.94 years old. Male patients accounted for 54.9%, HFpEF, HFmrEF and HFrEF patients accounted for 53.1, 24.8 and 22.1%, respectively. About 41.6% cases were HF accompanied with coronary artery disease, while 62.8% cases were HF combined with hypertension. Other HF patients combined with diabetes mellitus, atrial fibrillation accounted for 40.7, 45.1%, respectively. See Table 1.

**Table 1:** Basic data of HF patients

Project	Basic data
Age (years old)	69.92 ± 13.94
Male [Case (%)]	62 (54.9%)
Type of HF [Case (%)]	
HFpEF	60 (53.1%)
HFmrEF	28 (24.8%)
HFrEF	25 (22.1%)
Premedication history [Case (%)]	
Cardiotonic	36 (31.9%)
Diuretic	75 (66.4%)
Aldosterone receptor antagonist	31 (27.4%)
ACEI/ ARB	42 (38.1%)
β receptor blocker	32 (28.3%)
Calcium channel antagonist	40 (35.4%)
Aspirin/Clopidogrel	44 (38.9%)
Statins	46 (40.7%)
Complication [Case (%)]	
Coronary heart disease [Case (%)]	47 (41.6%)
Hypertension [Case (%)]	71 (62.8%)
Diabetes Mellitus [Case (%)]	46 (40.7%)
Atrial fibrillation [Case (%)]	51 (45.1%)
Smoke History [Case (%)]	28 (24.8%)

Note: *ACEI* Angiotensin Converting Enzyme Inhibitor, *ARB* Angiotensin Receptor Blocker

### General information of HF patients with different ejection fractions

Compared with indicators among HFpEF, HFmrEF and HFrEF groups, the results found that age, systolic blood pressure were statistically different among groups (*P* < 0.05), but there was no significant difference among three groups in gender, BMI, diastolic blood pressure, triglycerides, total cholesterol, Low density lipoprotein-cholesterol (LDL-C), the rate of each HF phenotype taking HF treatment (*P* > 0.05) and other indicators, see Table 2.

**Table 2:** Comparison of general data in HF patients with different ejection fractions

Projects	HFpEF group (≥50%, *n* = 60)	HFmrEF group (41–49%, *n* = 28)	HFrEF group (≤40%, *n* = 25)	*P* value
Age (year)	73.35 ± 11.31	68.82 ± 14.28	62.92 ± 16.79^a^	0.021
Male (case %)	26 (43.3%)	18 (64.3%)	18 (72.0%)	0.796
BMI (kg/m^2^)	23.95 ± 4.20	22.98 ± 3.03	23.03 ± 3.43	0.499
Systolic blood pressure (mm Hg)	139.28 ± 27.64	139.44 ± 22.51	121.04 ± 17.25^ab^	0.011
Diastolic blood pressure (mmHg)	76.07 ± 13.92	76.86 ± 14.76	78.20 ± 13.73	0.819
Serum sodium (mmol/L)	140.60 ± 3.95	140.36 ± 3.75	141.32 ± 3.02	0.607
Fasting glucose (mmol/L)	6.30 ± 1.11	6.54 ± 1.19	6.21 ± 0.97	0.504
LVEF (%)	57.19 ± 5.43	44.45 ± 2.99	33.23 ± 3.98	<0.01
Triglyceride (mmol/L)	1.39 ± 0.85	1.40 ± 0.74	1.38 ± 0.66	0.832
Total cholesterol (mmol/L)	4.04 ± 1.14	4.29 ± 1.24	4.25 ± 1.08	0.721
LDL-C (mmol/L)	2.54 ± 0.99	2.76 ± 1.08	2.85 ± 1.08	0.432
Serum Creatinine (μmol/L)	132.08 ± 20.63	108.71 ± 49.31	104.12 ± 44.78	0.813
eGFR (ml/min/1.73m^2^)	65.52 ± 3.94	55.99 ± 5.37	53.20 ± 8.48	0.237
ACEI/ARB [case (%)]	22 (36.7%)	13 (46.4%)	7 (28%)	0.383
β-blockers [case (%)]	18 (30.0%)	7 (25.0%)	7 (28.0%)	0.889
MRA [case (%)]	12(20.0%)	8(28.6%)	11(44.0%)	0.079

Note: *BMI* Body Mass Index, *LDL-C* Low Density Lipoprotein Cholesterol, *LVEF* Left Ventricular Ejection Fraction, *eGFR* Estimated Glomerular Filtration Rate, 1 mmHg = 0.133 kPa, a: Compared with HFpEF group, b: Compared with HFmrEF: *P<0.05

### Comparison of biomarker levels in HF patients with different ejection fractions

The levels of plasma sST2 in HFpEF, HFmrEF, HFrEF patients were 1.31 (0.30, 2.80), 1.31 (0.30, 2.80) and 5.26 (2.82, 7.56) ng/ml, respectively. The plasma MMP-2 levels in HFpEF, HFmrEF, HFrEF patients were 3.81 (2.50, 5.75), 4.01 (2.31, 8.85) and 7.68 (3.24, 7.56) ng/ml, respectively. The plasma MMP-9 levels of HFpEF, HFmrEF, HFrEF patients were 9.73 (3.69, 20.08), 11.39 (6.58, 26.10) and 22.43 (7.95, 32.46) ng/ml, respectively. The levels of plasma NT-proBNP in HFpEF, HFmrEF, HFrEF patients were 2346.50 (838.77, 8164.00), 4536.00 (921.90, 9220.00) and 5934.00 (2871.50, 15,520.50) pg/ml, respectively, with the decrease of ejection fraction, the levels of these biomarkers showed an upward trend and it presented a statistical significance (*P*<0.01).

**Fig. 1:**
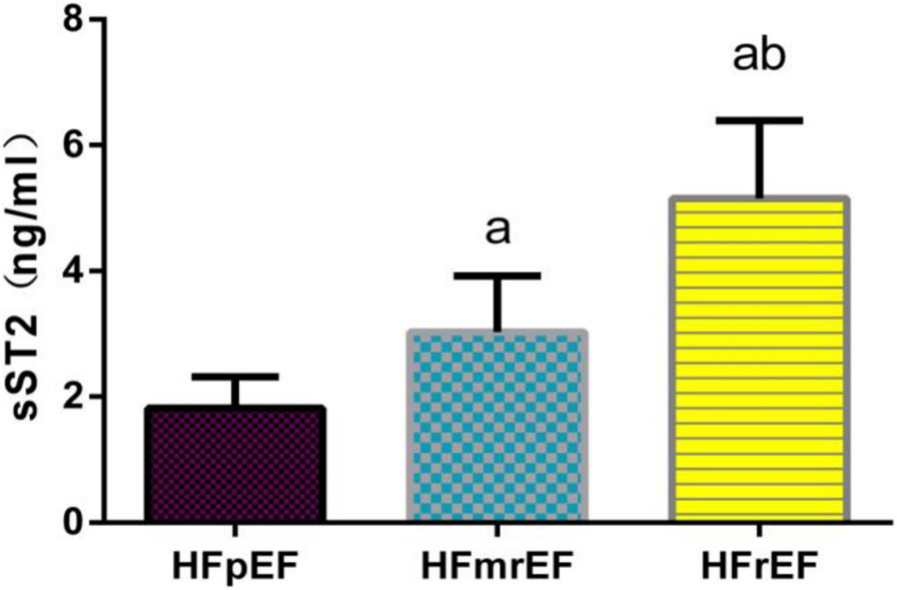
Comparison of plasma sST2 levels among groups. Note: a: Compared with HFpEF group *P*<0.01, b: Compared with HFmrEF group *P*<0.01

**Fig. 2:**
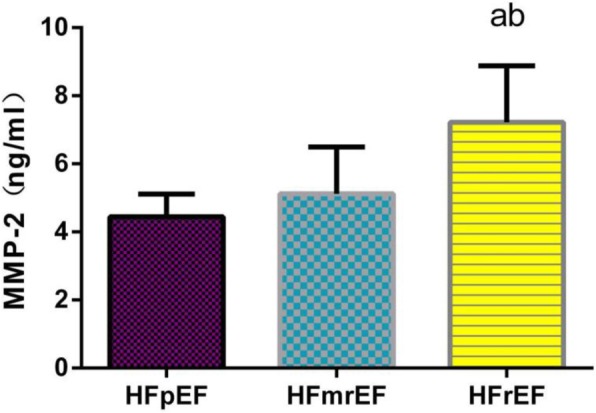
Comparison of plasma MMP-2 levels among groups. Note: a: Compared with HFpEF group *P*<0.01b: Compared with HFmrEF group *P*<0.01

**Fig. 3:**
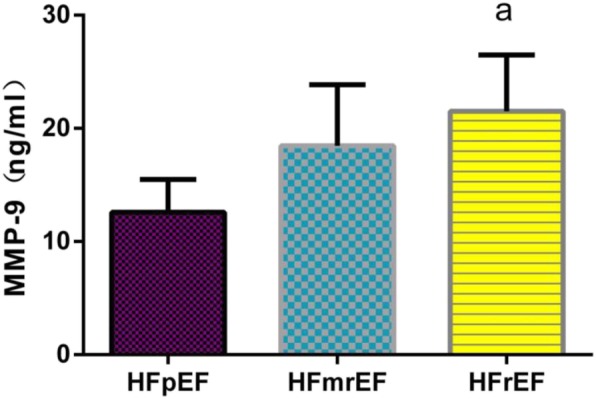
Comparison of plasma MMP-9 levels among groups. Note: a: Compared with HFpEF group *P*<0.01

**Fig. 4:**
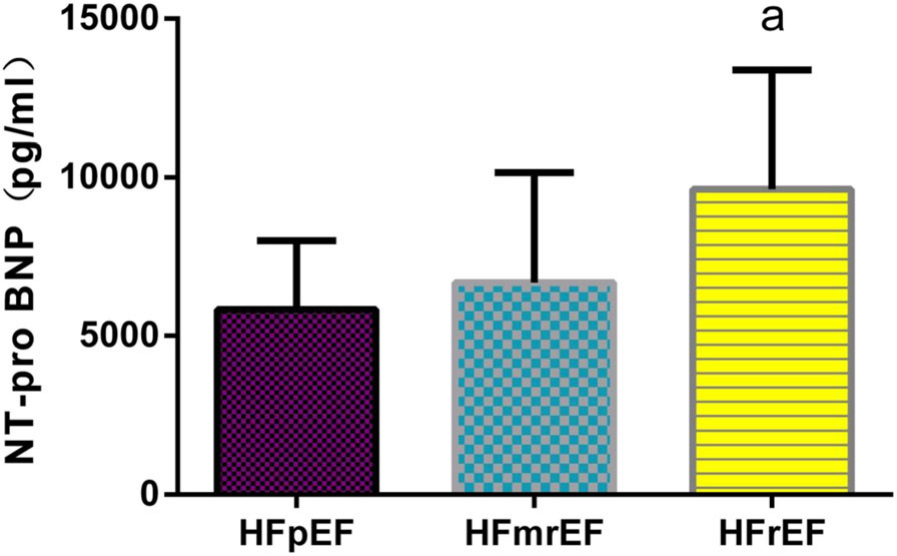
Comparison of plasma NT-proBNP levels among groups. Note: a: Compared with HFpEF group *P*<0.01

Further multiple comparisons of plasma sST2 levels among the groups revealed that there is significant difference between every two groups (*P* < 0.01), as shown in Fig. 1. The plasma MMP-2 levels were significantly higher in the HFrEF group than in HFmrEF group and the HFpEF group (*P* < 0.01), but there was no statistical difference between’ HFpEF and HFmrEF groups (*P* > 0.05), as shown in Fig. 2. The plasma MMP-9 and NT-proBNP levels were significantly higher in the HFrEF group than in the HFmrEF group (*P* < 0.01), but there is no statistical difference between HFpEF and HFmrEF group in MMP-9 and NT-proBNP levels (*P*>0.05), there is no statistical difference between HFrEF and HFmrEF group in MMP-9 and NT-proBNP levels either (*P*>0.05), as shown in Figs. 3 and 4.

### Diagnostic value of biomarkers in HFpEF patients

**Fig. 5:**
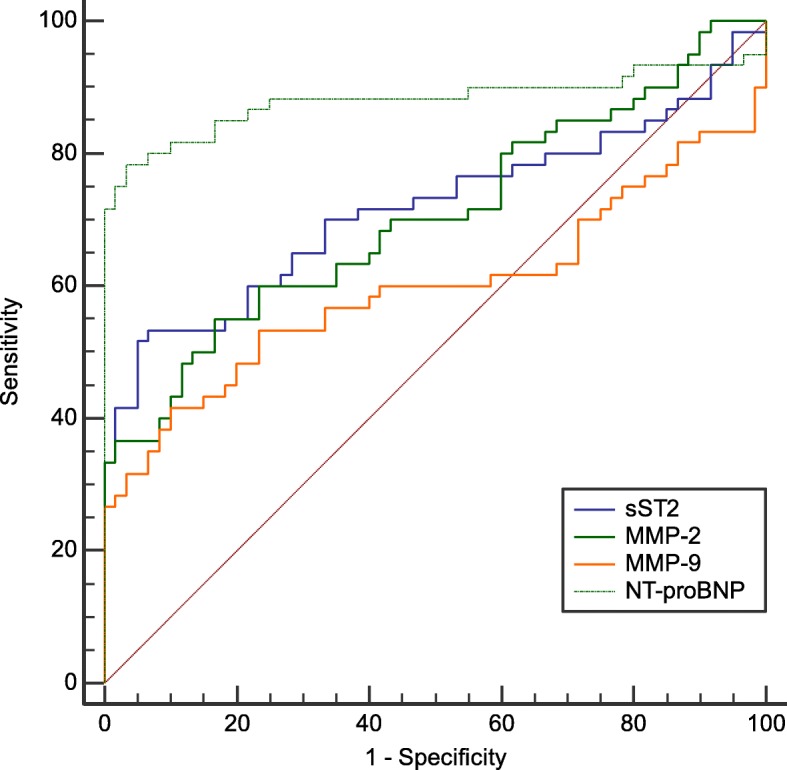
ROC curve of biomarkers plasma sST2, MMP-2, MMP-9 and NT-proBNP for diagnosis of HFpEF

In this study, the ROC curve was used to analyze the diagnostic value of plasma sST2, MMP-2, MMP-9 and NT-proBNP in HFpEF. The results suggested that NT-proBNP had a higher AUC for the diagnosis of HFpEF than sST2, MMP-2 and MMP-9, and its sensitivity and specificity were also higher. The AUC of plasma sST2 for diagnosis of HFpEF was 0.717 (95% confidence interval: 0.628–0.796, *P* < 0.01), the optimal cut-off value was 0.332 ng/ml, the sensitivity was 51.7%, and the specificity was 95%. The AUC of plasma MMP-2 for diagnosis of HFpEF was 0.705 (95% confidence interval: 0.615–0.785, *P* < 0.01). The optimal cut-off value was 3.138 ng/ml, the sensitivity was 55%, and the specificity was 83.3%. The AUC of plasma MMP-9 for diagnosis of HFpEF was 0.597 (95% confidence interval: 0.504–0.686, *P* < 0.01), the optimal cut-off value was 13.111 ng/ml, the sensitivity was 41.7%, and the specificity was 90%. The AUC of plasma NT-proBNP for diagnosis of HFpEF was 0.881 (95% confidence interval: 0.809–0.933, *P* < 0.01). The point value was 799.750 pg/ml, the sensitivity was 78.3%, and the specificity was 96.7%, as shown in Fig. 5.

### Diagnostic value of bio-markers in HFmrEF patients

**Fig. 6:**
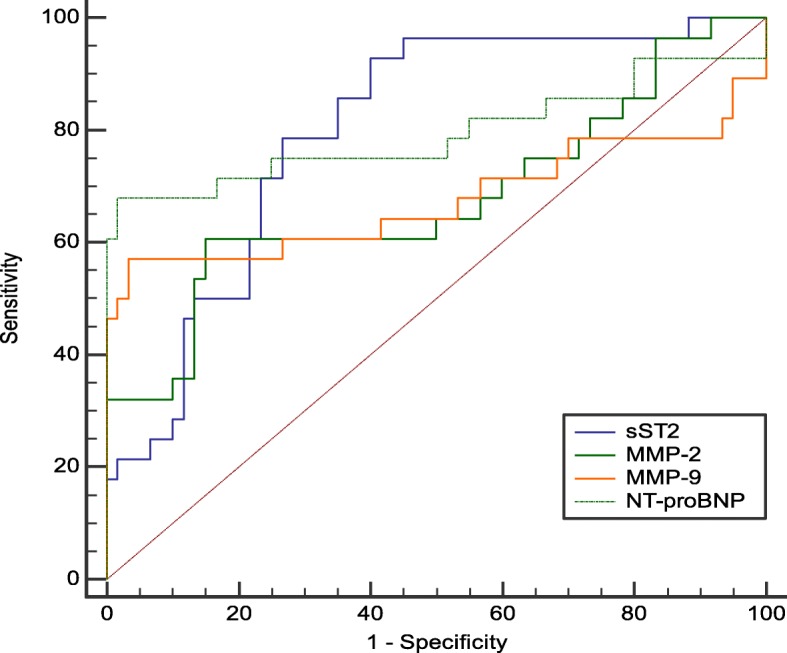
ROC curve of biomarkers plasma sST2, MMP-2, MMP-9 and NT-proBNP for diagnosis of HFmrEF

In this study, the ROC curve was used to analyze the diagnostic value of plasma sST2, MMP-2, MMP-9 and NT-proBNP in HFmrEF. The results suggested that sST2 had a higher AUC for the diagnosis of HFmrEF than MMP-2, MMP-9 and NT-proBNP, and its sensitivity and specificity were also higher. The results showed that the AUC of plasma sST2 for diagnosis of HFmrEF was 0.799 (95% confidence interval: 0.701–0.877, *P* < 0.01), the optimal cut-off value was 0.565 ng/ml, the sensitivity was 92.9%, and the specificity was 60.0%. The AUC of plasma MMP-2 for diagnosis of HFmrEF was 0.678 (95% confidence interval: 0.570–0.774, *P* < 0.01), the optimal cut-off value was 3.798 ng/ml, the sensitivity was 60.7%, and the specificity was 85%. The AUC of plasma MMP-9 for diagnosis of HFmrEF was 0.676 (95% confidence interval: 0.567–0.772, *P* < 0.01), the optimal cut-off value was 15.049 ng/ml, the sensitivity was 57.1%, and the specificity was 96.7%. The AUC of plasma NT-proBNP for diagnosis of HFmrEF was 0.793 (95% confidence interval: 0.694–0.872, *P* < 0.01), the cut-off value is 898.1 pg/ml, the sensitivity is 67.9%, and the specificity is 98.3%, as shown in Fig. 6.

### Diagnostic value of bio-markers in HFrEF patients

**Fig. 7:**
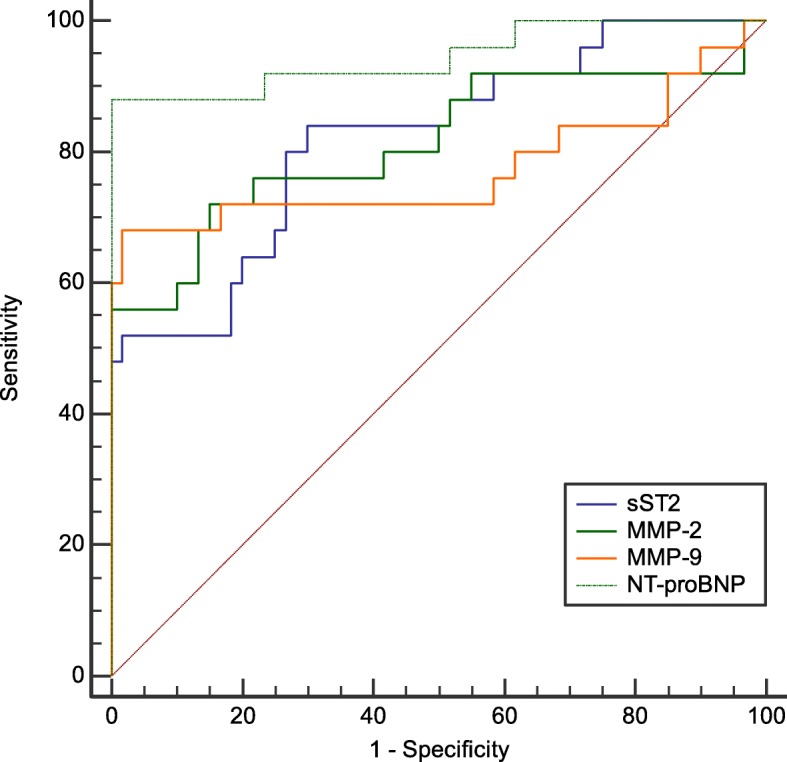
ROC curve of biomarkers plasma sST2, MMP-2, MMP-9 and NT-proBNP for diagnosis of HFrEF

In this study, the ROC curve was used to analyze the diagnostic value of sST2, MMP-2, MMP-9 and NT-proBNP in HFrEF. The results suggested that NT-proBNP had a higher AUC for the diagnosis of HFrEF than sST2, MMP-2 and MMP-9, and its sensitivity and specificity were also higher. The results showed that the AUC of plasma NT-proBNP for diagnosis of HFrEF was 0.945 (95% confidence interval: 0.874–0.983, *P* < 0.01), the cut-off value was 1106.700 pg/ml, the sensitivity was 88.0%, and the specificity was 98.3%. The AUC of plasma sST2 for diagnosis of HFrEF was 0.820 (95% confidence interval: 0.722–0.895, *P* < 0.01), the optimal cut-off value was 2.539 ng/ml, the sensitivity was 84.0%, and the specificity was 70.0%. The AUC of plasma MMP-2 for diagnosis of HFrEF was 0.814 (95% confidence interval: 0.715–0.890, *P* < 0.01), the optimal cut-off value was 3.846 ng/ml, the sensitivity was 72.0%, and the specificity was 85.0%. The AUC of plasma MMP-9 for diagnosis of HFrEF was 0.774 (95% confidence interval: 0.670–0.858, *P* < 0.01), the optimal cut-off value was 16.748 ng/ml, the sensitivity was 68.0%, and the specificity was 98.3%, as shown in Fig. 7.

### The relationship of plasma sST2 levels and related factors in HF patients with different ejection fractions

Spearman correlation analysis results suggest that the plasma sST2 levels are positively correlated with plasma MMP-2 (r value were + 0.330, + 0.505, + 0.562 respectively, *P*<0.05), MMP-9 (r value were + 0.558, + 0.376, + 0.811, *P*<0.05) and NT-proBNP (r value were + 0.493, + 0.448, + 0.688, *P*<0.05), regardless of the value of LVEF, as shown in Table 3.

**Table 3:** Spearman correlation analysis of plasma sST2 levels and related factors in patients with HF

Projects	HFpEF group (n = 60)		HFmrEF group (n = 28)		HFrEF group (n = 25)	
–	*r*	*P*	*r*	*P*	*r*	*P*
Age (Years old)	+ 0.058	0.685	−0.087	0.660	+ 0.106	0.614
Male [Case (%)]	+ 0.185	0.276	+ 0.240	0.219	−0.046	0.670
BMI (kg/m^2^)	+ 0.028	0.833	−0.087	0.660	−0.055	0.792
Diabetes Mellitus [Case (%)]	+ 0.191	0.075	−0.118	0.462	+ 0.059	0.727
Atrial fibrillation [Case (%)]	+ 0.087	0.415	+ 0.100	0.553	+ 0.277	0.103
Coronary heart disease [Case (%)]	+ 0.102	0.341	−0.136	0.394	+ 0.062	0.717
Hypertension [Case (%)]	+ 0.120	0.263	−0.132	0.441	+ 0.289	0.089
Systolic blood pressure (mmHg)	−0.005	0.969	+ 0.048	0.808	+ 0.037	0.861
Diastolic blood pressure (mmHg)	−0.014	0.913	+ 0.355	0.063	+ 0.105	0.618
Triglyceride (mmol/L)	+ 0.05	0.705	−0.101	0.609	−0.072	0.733
Total cholesterol (mmol/L)	−0.132	0.314	+ 0.252	0.196	−0.251	0.226
LDL-C (mmol/L)	−0.090	0.496	+ 0.360	0.060	−0.231	0.132
eGFR [ml/(min·1.73m^2^)]	−0.117	0.726	+ 0.008	0.969	−0.195	0.351
MMP-2 (ng/ml)	+ 0.330	0.010	+ 0.505	<0.05	+ 0.562	0.003
MMP-9 (ng/ml)	+ 0.558	<0.05	+ 0.376	0.001	+ 0.811	<0.05
NT-proBNP (pg/ml)	+ 0.493	<0.05	+ 0.448	<0.05	+ 0.688	<0.05

Note: BMI: body mass index, LDL-C: low density lipoprotein cholesterol, eGFR: estimated glomerular filtration rate

### The analysis of affecting factors with HF

HF was taken as a dependent variable, while age, gender, LVEF, blood lipids, blood pressure, BMI, creatinine, sST2, MMP-2, MMP-9, and NT-proBNP were gradually entered into the model as an independent variable. The significance level was defined as 0.05 after the model was selected. Logistic regression analysis was performed after excluding the confounding factors and interaction effect. The results showed that the independent variables such as age, sST2, MMP-2 and NT-proBNP were independent risk factors for heart failure, as shown in Table 4.

**Table 4:** Logistic regression analysis of factors affecting HF

Projects	*OR*	*Wald*	95% *CI*	*P* value
Age	0.140	10.384	1.056–1.252	0.001
sST2 (ng/ml)	1.960	3.797	1.409–35.758	0.004
MMP-2 (ng/ml)	0.805	5.468	1.139–4.390	0.019
NT-proBNP (pg/ml)	0.002	4.412	1.001–1.004	0.036

Note: *OR* Odds ratio, *CI* confidence interval

### Total end-point events of HF during follow-up

There were 113 subjects were enrolled in this study, six of them were lost to follow up, the loss ratio was 5.3%. 32 patients were dead during a median follow-up of 778 days (CI: 540 days–1080 days). In our study, the all-cause mortality rate was 29.9%, and rate of re-admission to HF was 38.3%, incidence of combined endpoints was 53.2%. In this study, re-admission rate of patients with HFpEF, HFmrEF, and HFrEF during a median follow-up of 778 days were 23.3, 39.2 and 56.0% respectively, and the incidence rate of combined end points were 23.3, 56.2, and 75.1%, respectively. All these data were statistically significant (*P* < 0.05, as shown in Table 5).

**Table 5:** Main endpoint of HFpEF, HFmrEF and HFrEF patients during a median follow-up of 778 days

Endpoint (%)	HFpEF	HFmrEF	HFrEF	*P*
All-cause mortality	16.6	35.7	48.0	0.124
Heart failure readmission	23.3	39.2	56.0	0.013
Combined endpoint	31.3	56.2	75.1	0.008

Note: Combined end point: all-cause mortality and/or re-admission of HF

### Effect of plasma sST2 baseline on endpoint events

**Fig. 8:**
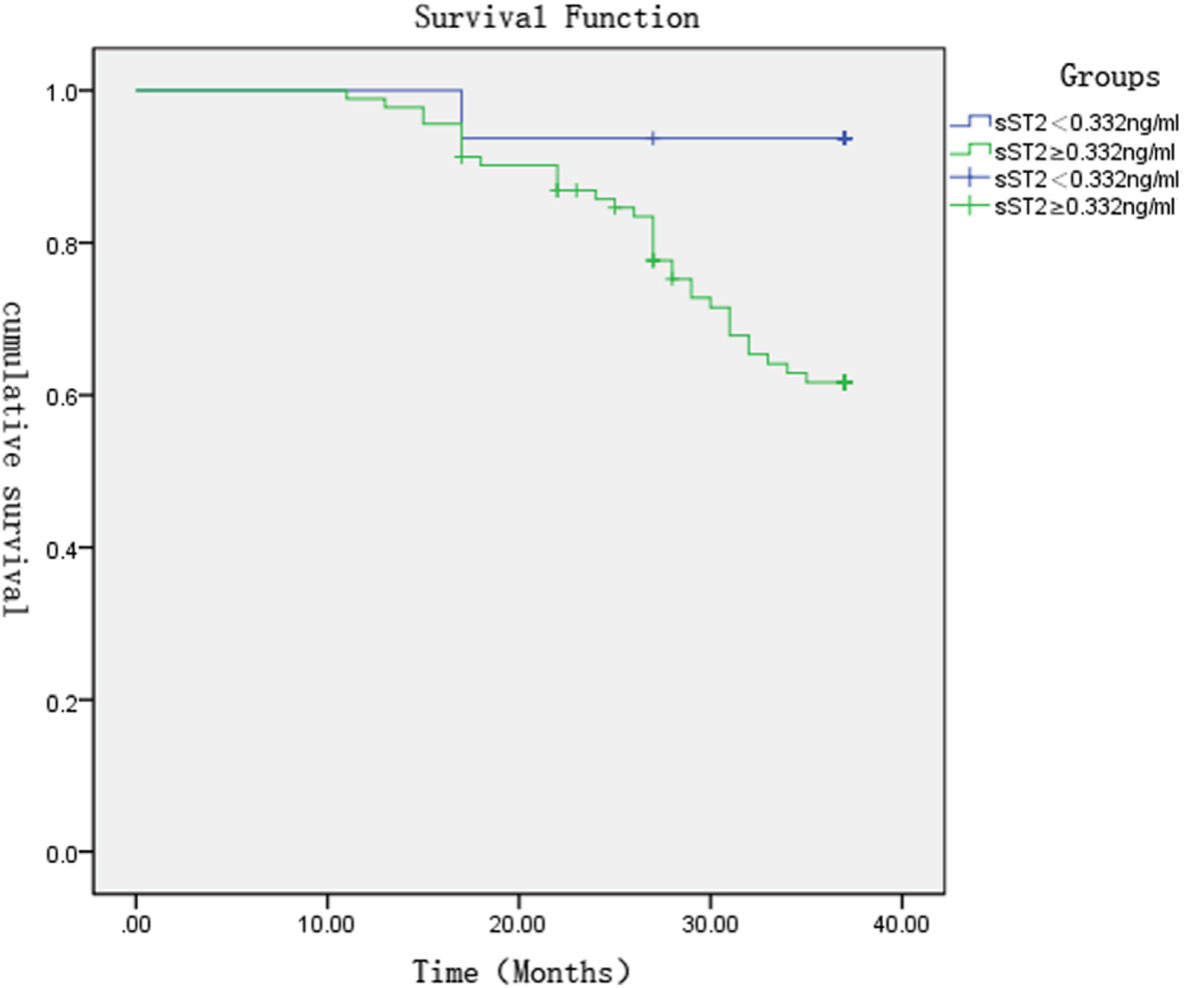
Survival analysis of sST2 baseline levels at admission

We compared the baseline of plasma sST2 to find its relationship with the risk of adverse events: re-admission or all-cause death to HF during a median follow-up of 778 days after discharge. The patients were divided into low-level group (sST2 < 0.332 ng / ml) and high-level group (sST2 ≥ 0.332 ng / ml) according to the value of sST2 which the optimal cut off value of plasma sST2 at admission was obtained through the ROC curve analysis of Fig. 8. Survival analysis indicated that the patients in the high-level sST2 group suffered from a higher risk of end-point events and a lower survival rate (log-rank chi-square value = 5.036, *P* = 0.025), as shown in Table 6 and Fig. 8.

**Table 6:** Comparison of survival status under plasmas ST2 baseline levels

Indicator		Survival rate	Mortality rate	c^2^	*P*
sST2(ng/ml)	<0.332	93.7%	6.3%	5.036	0.025
	≥0.332	64.1%	35.9%		

## Discussion

HF is the end stage of several pathological cardiac conditions and is currently the most common cause of death in cardiovascular diseases. Nowadays, the guidelines of HF in various countries mainly recommend brain natriuretic peptide (BNP) and NT-proBNP [2] to be a useful biomarker in the judgment of diagnosis, severity degree assessment, and prognostic evaluation of endpoint events for HF. Plasma NT-proBNP level is more sensitive than BNP when cardiac pressure or volume load increases, the half-life of NT-proBNP is also longer and more stable [7]. However, NT-proBNP can’t correctly reflect degree of left ventricular filling because the relatively longer half-life can be affected by the retention of fluid in the body [3]. Besides, NT-proBNP level was affected by many confounding factors, such as age, LV hypertrophy, renal insufficiency and dyspnea which frequently lead to false negative results and the wrong clinical judgment. At present, there is no ideal biomarker can be used for the early diagnosis, the severity and prognosis of HF and not affected by other non-cardiac factors.

It’s reported that Trans mural stress stimulation could cause an increase in ventricular wall tension, thus, cardiomyocytes were mechanically stretched and stimulated to secrete NT-proBNP, as well as the sST2, MMP-2 and MMP-9. In the MOCA study, various biomarkers such as sST2, BNP, and NT-proBNP were evaluated, and they were all identified as independent risk factors [8]. Our study found that regardless of the level of LVEF, the sST2 levels in patients at admission were positively correlated with MMP-2, MMP-9 and NT-proBNP. sST2, MMP-2, and NT-proBNP were all independent risk factors of heart failure which can be used for risk stratification. This may be related to age-related vascular stiffness and intensified left ventricular wall tension. Recently, biomarkers related to myocardial remodeling, such as sST2 and MMPs, have been recommended by the international HF guidelines in the diagnosis and prognosis of HF. However, there is neither Asian report about their application, nor reports of large data in patients with HFmrEF. Therefore, in our study, we focused on the NT-proBNP, sST2 and MMPs, and compared the diagnostic and prognostic value of these biomarkers in HF patients with different ejection fraction for precision diagnosis of HF.

SANDERS-VAN et al. [9] scholars found that patients with HFrEF had higher levels of plasma NT-proBNP than patients with HFpEF, and the correlation in HFrEF was also higher than in HFpEF. This may owe to lower left ventricular pressure in HFpEF patients. Our study observed that the plasma NT-proBNP concentration gradually increased with the decrease of LVEF, besides, plasma NT-proBNP levels in HFrEF patients were significantly higher than in HFpEF patients, which is consistent with previous studies [10]. In the diagnostic value assessment of the four biomarkers for three types of heart failure, the AUC for the diagnosis of HFpEF and HFrEF by plasma NT-proBNP is higher than that of plasma sST2, MMP-2, and MMP-9, suggesting that the diagnostic predictive value of plasma NT-proBNP for HFpEF and HFrEF is better than that of other markers.

Cardiac collagen remodeling is important in the progression of heart failure. Serum markers of cardiac extracellular matrix (ECM) turnover proteins such as MMPs are positively correlated with interstitial fibrosis, diastolic dysfunction and left ventricular hypertrophy [10, 11]. MMP-2 and MMP-9 are important components of ECM. In a study of 62 patients with chronic HF, MMP-2 levels were reduced in patients with significant LVEF recovery after treatment with β blockers, while MMP-2 levels were elevated in patients with less improved LVEF [12]. Our results showed that the plasma levels of MMP-2 and MMP-9 were significantly higher in the HFrEF group than in HFmrEF and HFpEF group (*P* < 0.01), but there was no statistical difference between HFpEF and HFmrEF groups (*P* > 0.05). Further Spearman correlation analysis suggested that the MMP-2 but not MMP-9 could be used as an independent risk factors for heart failure diagnosis, which was consistent with the results of George J’s study [13]。.

ST2 is a member of the interleukin 1 receptor family and exists in two forms, a trans-membrane receptor (ST2L) as well as a soluble decoy receptor (sST2) [14]. The ligand of ST2 is Interleukin-33 (IL-33), which is involved in reducing fibrosis and hypertrophy in mechanically strained tissues. Overexpression of sST2 was significantly related to poor myocardial remodeling, cardiac insufficiency, and hemodynamic abnormalities [15]. Tseng et al. found that the patients in the end-stage of HF had a higher sST2 level compared with the patients in the NYHA stage II and III of HF, but the level could decrease within 3 months after implantation of left ventricular assist device [16]. Shah et al. [17] found that sST2 levels were significantly associated with abnormal changes in function and structure such as ventricular enlargement, LV diastolic dysfunction etc. In our study, Spearman correlation analysis results indicated that sST2 could be an independent risk factor for heart failure diagnosis. Besides, we found there is a statistical difference when compared the sST2 levels in HFmrEF and HFpEF patients, the area under the ROC curve for the diagnosis of HFmrEF by plasma sST2 levels is significantly higher than that of plasma MMPs and NT-proBNP, and its sensitivity and specificity are both higher, suggesting that the independent diagnostic value of plasma sST2 levels for HFmrEF is better than that of plasma NT-proBNP and MMPs. It can be read that sST2 is a more accurate marker for the diagnosis of HFmrEF in the “grey zone” of heart failure patients, which is also consistent with previous studies [18]. Further survival analysis of our study indicated that the HF patients in the base high-level sST2 suffered from a higher risk of end-point events and a lower survival rate. This result proved the same as Vark’s study. We can read that in terms of prognostic value of HF, sST2 is considered to be a better predictor than NT-proBNP and MMPs [19].

Since this study did not analyze indicators associated with diastolic function in echocardiography, it is necessary to further study related factors in the subsequent studies.

## Conclusion

The markers sST2, MMP-2 and NT-proBNP are all independent risk factors for patients with heart failure. The plasma sST2 levels can be a useful marker with both diagnostic and prognostic values in patients with HFmrEF. The higher the plasma sST2 level in patients with heart failure was related to a higher incidence of combined endpoint outcome.

## Data Availability

The datasets used and/or analyzed during the current study are de-identified and available from the corresponding author on reasonable request.

## Abbreviations

NT-proBNP: N-terminal of the prohormone brain natriuretic peptide
sST2: Soluble Supperssion of tumorigenicity 2
MMPs: Matrix metalloproteinases
HF: Heart failure
HFpEF: HF with preserved ejection fraction
HFmrEF: HF with mid-range ejection fraction
HFrEF: HF with reduced ejection fraction
ELISA: Enzyme linked immunosorbent assay
ROC: Receiver operating characteristic
AUC: The area under the curve
OR: Odds ratio
BMI: Body mass index
LV: Left ventricular
LVEF: LV ejection fraction
EDTA: Ethylene Diamine Tetraacetic acid
ACEI: Angiotensin converting enzyme inhibitors
ARB: Angiotensin receptor blocker
LDL-C: Low density lipoprotein-cholesterol
eGFR: Estimated glomerular filtration rate
ECM: Extracellular matrix
IL-33: Interleukin-33
CI: Confidence interval
BNP: Brain natriuretic peptide
